# The trust–NPS correlation: The role of trust in promoting customer loyalty in Swiss financial institutions

**DOI:** 10.1371/journal.pone.0334423

**Published:** 2025-11-05

**Authors:** Vanessa Gabriela Yarza Navarro-Schär, Eric Eller, Marco Schulz, Eva Lermer

**Affiliations:** 1 Center for Leadership and People Management, Ludwig-Maximilians-Universität München, Munich, Germany; 2 Business School, Technische Hochschule Ingolstadt, Ingolstadt, Germany; 3 elaboratum Suisse, Zurich, Switzerland; 4 Department Business Psychology, Technische Hochschule Augsburg, Augsburg, Germany; Heriot-Watt University, UNITED KINGDOM OF GREAT BRITAIN AND NORTHERN IRELAND

## Abstract

The Net Promoter Score (NPS) is a widely used key performance indicator (KPI) for measuring customer loyalty and guiding customer-centric strategies. While customer trust has been identified as an important influencing factor for NPS, existing research treats trust as a unidimensional construct, leaving the specific trust components that drive customer loyalty behaviors largely unexplored. This study addresses this theoretical gap by examining the relationship between various trust components and NPS in the financial services sector. A survey of 1,370 Swiss consumers assessed trust across ten theoretically-derived dimensions alongside their NPS ratings for five financial service companies. Employing median-split analysis, results demonstrate that high customer trust is associated with a significantly higher NPS (+36.5) compared to low trust (−68.6). Critically, quantile regression analysis revealed heterogeneous effects of trust components across different NPS distribution levels, with ability, experience, and reputation showing consistent positive effects across all quantiles. In contrast, joint interests and continuity emerged as relevant only at higher NPS quantiles, while reciprocity proved significant exclusively at the 25th percentile, demonstrating that distinct trust determinants become salient depending on customers’ advocacy levels. These findings challenge prevailing theoretical assumptions about customer trust’s homogeneous influence on customer loyalty, revealing instead a differentiated pattern of trust component effects. The study advances trust theory by demonstrating that trust dimensions not only vary in their influence on customer advocacy behaviors but also exhibit different effect patterns across the NPS distribution, contradicting assumptions of uniform trust impact on loyalty outcomes. The results indicate that NPS optimization requires strategic focus on specific trust dimensions, particularly those demonstrating consistent positive associations across quantile levels. This research contributes novel theoretical insights into the conditional trust-loyalty relationship while providing empirically-grounded guidance for customer-centric business strategies.

## Introduction

How can companies effectively foster customer loyalty in an increasingly competitive landscape? This question has become central to business strategy [1], as customer retention is often more cost-effective than acquisition [2], and loyal customers tend to generate higher lifetime value [3]. Brand loyalty serves as a critical differentiator that enables companies to build resilient customer relationships, reduce marketing costs, and maintain competitive advantage through stronger emotional connections and shared values with consumers [4]. Despite investing heavily in customer experience programs, many companies see their NPS scores stagnate or even decline, often because they focus on surface-level improvements rather than addressing the fundamental psychological drivers of loyalty.[5]. The Net Promoter Score (NPS) has emerged as the predominant metric for measuring customer loyalty and advocacy intentions, captured through the simple question: “How likely is it that you would recommend this organization to a friend or colleague?” [6–8]. Based on an 11-point Likert scale [5,9], customers are categorized as promoters (9–10), passives (7–8), and detractors (0–6), with the final NPS calculated by subtracting the percentage of detractors from promoters, resulting in a score between −100 and +100 [10]. NPS gained popularity for its operational simplicity and its strong correlation with business growth and profitability outcomes [11].

The metric helps companies focus on customer-centricity and customer relationships [12] and thereby find improvement potential, thus driving growth [13]. However, despite widespread corporate initiatives to enhance NPS, organizational approaches typically emphasize generalized strategic interventions rather than systematically addressing the specific psychological determinants that drive customer advocacy behaviors [14,15]. Among the various factors influencing NPS, empirical evidence increasingly demonstrates trust as a predominant predictor of customer recommendation likelihood [8,10,11]. However, organizations frequently lack structured methodologies for cultivating and evaluating customer trust relationships. Given trust’s multidimensional nature, research has concentrated on deconstructing and operationalizing its constituent elements [16,17].

From a practical perspective, understanding the differential impact of specific trust components on NPS is essential for organizations seeking to optimize their trust-building investments and resource allocation [18]. Rather than implementing broad trust enhancement strategies, companies require empirically-grounded guidance on which trust dimensions yield the greatest returns in terms of customer advocacy behaviors [19]. This knowledge enables managers to prioritize specific trust-building activities and develop targeted interventions that maximize NPS improvements within constrained budgets and timeframes. Although trust has been identified as an important influencing factor for customer loyalty [9], the specific relationship between trust components and NPS remains largely unexplored [20]. By examining these relationships empirically, research can begin to develop more precise theoretical models that explain how different trust mechanisms influence customer advocacy behaviors.

The NPS is a pragmatic measure of customer loyalty that captures the likelihood of customers recommending a product or service [10]. NPS supports customer centricity by seeking direct feedback and thus understanding customer satisfaction, behavior, and needs to enable customer-focused strategies [2,15]. This helps companies gauge customer value and loyalty, leading to increased overall performance [12]. Companies strive to improve NPS by tracking experiences and influential factors [21,22]. Insights from business psychology can improve NPS by uncovering its psychological determinants [15]. While multiple factors influence NPS, mounting evidence suggests that customer trust serves as a particularly powerful predictor – yet most companies lack a systematic approach to building and measuring this trust [20,23,24]. Since trust is complex, efforts have focused on measuring and managing its components [17,25].

### Trust as a key driver for NPS

Customer trust has been identified as a key factor influencing the Net Promoter Score (NPS), although a deeper understanding of how trust manifests for businesses is needed [8]. Previous researches has confirmed that a high NPS is significantly influenced by customer trust, and its absence leads to increased customer attrition [6,11,13,26]. However, NPS, as a proxy for customer loyalty, is not a monolithic construct and should be interpreted within a broader socio-psychological and institutional context [27]. Despite the recognized positive influence of customer trust on NPS [13], the interaction of specific trust components with NPS remains unclear [20]. Various trust models (e.g. the model of trust [28], the web-trust model [29], the conceptual model [30] and the bank system trust [31]) suggest various trust components [29]. Early foundational work operationalized trust through five dimensions: benevolence, integrity, ability, expertise, shared values, and communications [32]. Factor analysis revealed that shared values emerged as the weakest component while benevolence and integrity showed the strongest correlations with overall trust [32]. Subsequent research refined these dimensions, finding that integrity and consistency emerged as the strongest predictor of trust, followed by concern and benevolence. Communications and experience showed moderate effects, while shared values consistently demonstrated the weakest relationships with trusting beliefs [30]. Longitudinal research examining trust trends has revealed important variations across provider types and time periods. Trust measurements using cognitive and affective dimensions showed significant differences between institution types, with some providers achieving positive trust scores while others recorded negative values [33]. These studies found that transparency and fair treatment were strong predictors of narrow-scope trust, while broader institutional factors influenced system-level trust perceptions [32]. Comprehensive testing of six trust determinants – competence, consistency, integrity, customer orientation, clarity, and value congruence – through structural equation modeling revealed integrity as the most important determinant, followed by transparency, customer orientation, and competence [31]. Notably, stability and value congruence showed no significant effects on trust, challenging assumptions about financial stability perceptions. System trust was found to positively influence institution trust, supporting institutional theory predictions [32].

These empirical findings reveal several patterns: integrity consistently emerges as critical across different methodologies, transparency generally proves more important than value alignment, and institutional-level components provide important contextual foundations [28,30,34]. When adopting a broader perspective on the concept of customer trust, specific trust dimensions can be identified that are presumed to exert a potentially positive influence on the Net Promoter Score (NPS). These includeability (i.e., competence in fulfilling promises [35,36]), benevolence (i.e., goodwill of the trust taker towards the trust giver [37]), clarity (i.e., high-quality, unambiguous information [38,39]) consequences (i.e., trustworthy behaviors are rewarded [35,40]), continuity (i.e., consistency of promised behavior [30,35]); experience (i.e., previous interaction with the trust recipient [41,42]), integrity (matching words and deeds [43,44]) joint interest (i.e., alignment of interests between both parties [45,46]), reputation (i.e., positive evaluations from relevant others [35,47]), and reciprocity (i.e., mutual vulnerability [48,49]).

This study explores how these specific trust components affect NPS, a key performance indicator for companies [12,22]. For this purpose, an exploratory survey assessed trust in financial service companies and their NPS.

## Materials and methods

The trust-NPS correlations was investigated by examining consumer perceptions of five Swiss financial companies, amongst three insurers and two banks, using measures of customer trust and the NPS. The sampling mirrored the Swiss demographic distribution, recruiting current, past, and prospective Swiss consumers. The respondents were randomly assigned to one of the five companies for which they subsequently filled out a questionnaire. The recruitment period for this study took place from May 3, 2023 to May 5, 2023. At the beginning of the questionnaire, informed consent was obtained. Participants were informed that the data would primarily be collected for economic purposes, but could also be used for academic purposes. Consent was obtained by clicking the “continue” button. A total of *N* = 1,370 participants were recorded, with incomplete questionnaires or failed attention checks automatically filtered out by the panel. Of the respondents, 55.5% identified themselves as female, 44.2% as male, and 0.4% as another gender. The age distribution ranged from 19 to 70 years, with *M* = 40.83 years (*SD* = 15.35). A total, 37.0% of customers, 8.7% of former customers, and 54.3% of non-customers were surveyed.

Customer trust was measured regarding the ten trust components introduced above. To systematically measure these trust components, a comprehensive literature review of peer-reviewed questionnaires measuring trust between customers and companies was conducted. This systematic search yielded 670 items from 32 published questionnaires. These existing items were systematically evaluated and adapted to create a novel measurement instrument that comprehensively captures all ten theoretically grounded trust components relevant to this study’s research context. The literature review revealed varying levels of established measurement approaches across the ten trust components, with multiple validated items available for some dimensions (benevolence, integrity, interest alignment, clarity) while others required new item development based on theoretical definitions (consistency, reciprocity). Through rigorous scale development and testing procedures in the preliminary study, initial item pools were systematically refined to ensure optimal psychometric properties. The validation process involved reliability analysis using Cronbach’s alpha and factor analytical procedures, systematically eliminating items with poor psychometric properties while maintaining content coverage. All final subscales, reduced to four items each, demonstrated satisfactory reliability (*αs* ranging from .68 to.92; see S2 Table). Additionally, content validity was assessed using the Content Validity Ratio (CVR) [50] and its modification [51], with eight experts from academia and practice evaluating item appropriateness on a three-point scale, resulting all items achieving a CVR value > .75, indicating adequate content validity. Each trust component was measured using these four items on a 6-point Likert scale (see S1 Table). NPS was measured using the standard item described above, on a likert scale from 0–10 [10]. The questionnaire ended with the collection of demographic data and an attention check, which automatically filtered out inattentive participants for data quality. Further dependent variables like purchase intent and willingness to pay were surveyed using single-item scales. However, disregarded in this study’s parameters, they do not contribute to the research question.

To examine how different levels of customer trust affect NPS, a comprehensive analytical approach comprising four complementary methods was employed. Firstly, an aggregate score derived from all the trust components was presented as a representative measure of customer trust. Next, the sample was split into two distinct segments defined by high and low levels of customer trust, using the median split. This median split approach was chosen because it aligns with the theoretical understanding of trust as a threshold concept in consumer behavior, where the practical distinction between “high trust” and “low trust” states is more relevant for managerial decision-making than continuous gradations. Furthermore, the median split approach is particularly suitable for Likert scale data which may not meet the assumptions of normality required for parametric analyses, and it provides clear interpretability for practitioners seeking actionable insights from trust research [52]. The NPS was then calculated for each classification and a comparative analysis carried out.

In a second step, to understand the significant differences between the trust groups, a Mann-Whitney-U-test was employed to examine whether high and low trust segments differed sifgnificantly in their NPS evaluations. Given the use of the median as the primary statistic for group formation and the potential non-normal distribution of Likert scale data, the nonparametric Mann-Whitney-U test was more appropriate than parametric alternatives such as the independent t-test. The Wilcoxon test family is more suitable for analyzing data that do not follow a normal distribution and is less affected by outliers compared to parametric tests.

Thirdly, to gain a more comprehensive understanding of the relationship between customer trust components and NPS, a Spearman correlation analysis was additionally conducted. While the Mann-Whitney-U test provides information regarding group differences, correlation analysis offers insights into the strength and direction of associations between continuous variables, thereby enabling a more nuanced examination of the linear relationships and effect magnitudes between trust components and customer loyalty measures.

Finally, the relationship between the trust elements and the NPS was examined using quantile regression. Quantile regression was employed instead of ordinary least squares (OLS) regression to provide a more comprehensive understanding of the relationship between variables by examining different segments of the distribution rather than focusing solely on the mean. Specifically, the 25th, 50th (median), and 75th percentiles were analyzed to capture the full distributional relationship between trust components and NPS. This approach is particularly valuable when dealing with survey data and Likert scale measurements, as it is more robust to outliers and skewed distributions that commonly occur in such data. While parametric methods such as OLS regression are frequently used, quantile regression methods are more appropriate for datasets derived from surveys and provide insights into how trust-NPS relationships may vary across different levels of customer satisfaction. The implementation of these statistical methodologies contributes to enhanced analytical robustness and precision by providing insights into the differential impact of trust components upon customer loyalty across various segments of the NPS distribution. Histotrophic examination of the NPS variable revealed a non-normal and asymmetric distribution, suggesting that a conditional mean model may prove insufficient for adequately capturing the relationship between predictor variables and the criterion variable.

### Ethics statement

This study was conducted in accordance with the ethical principles outlined in the Declaration of Helsinki and the Belmont Report. Following institutional guidelines at the *Institute for Personality and Communication* in Munich, formal ethics committee approval was not required for this research, as it constituted a low-risk study involving anonymous survey data collection without any clinical interventions, sensitive health information, or vulnerable populations. The study involved prospective recruitment of human participants through the Bilendi Respondi online panel. All participants provided informed written consent through an electronic consent process prior to participation. Specifically, participants were presented with comprehensive information about the study’s purpose, procedures, voluntary nature of participation, and potential uses of the data for both economic and academic purposes. Informed consent was documented through participants’ affirmative action of clicking the “continue” button after reviewing the consent information. No minors were included in the study, and therefore parental or guardian consent was not applicable. Throughout the entire study period, no identifying information was collected from participants. Data collection was strictly limited to demographic variables (age and gender) for sample characterization purposes and study-relevant measures. The research design ensured complete participant anonymity, with no possibility for researchers to identify individual participants during or after data collection. All data were processed and analyzed exclusively in aggregate form, ensuring full compliance with privacy protection standards and eliminating any risk of individual identification. The study did not involve vulnerable populations, clinical trials, medical interventions, or collection of biological samples. No participants under the age of 18 or individuals requiring special ethical protections were included in the research.

## Results

**Fig 1:**
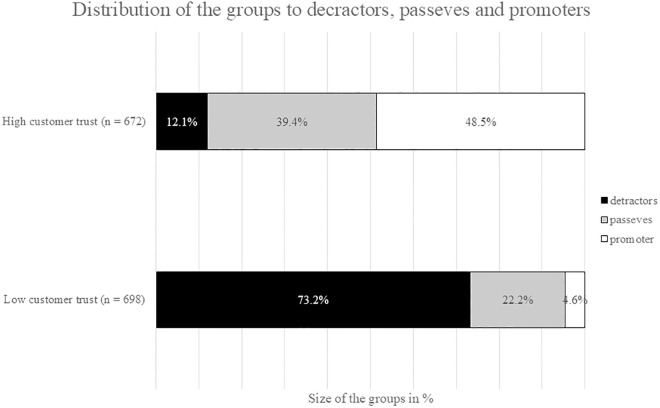
The distribution across the three NPS categories. Detractors, passives, and promoters were assessed for both the group of low trust customers and high trust customers. The floating values depicted above each bar symbolize the proportional representation of each category within its respective group.

To test if NPS is driven by customer trust, the sample was split into two equivalent groups, one with high customer trust and the other with low trust. Customers with low trust were predominantly represented in the detractor category (detractors = 73.2%; passives = 22.2%; promoters = 4.6%). Conversely, customers with high trust levels were primarily found within the Passive and Promoter categories (detractors = 12.1%; passives = 39.4%; promoters = 48.5%; see Fig 1). To elucidate the magnitude of association between customer trust and NPS, a Spearman correlational analysis was performed. Trust and NPS exhibited a statistically significant positive correlation (*r* = .789, *p* < .001, *N* = 1370). The observed correlation coefficient indicates that elevated trust levels are associated with higher NPS values, thereby suggesting a strong positive linear relationship between these constructs. This correlation magnitude represents a large effect [53].

**Fig 2:**
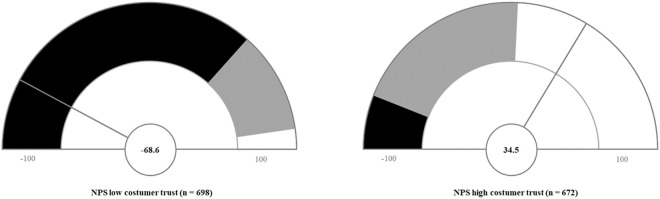
NPS for the groups of low customer trust and high customer trust. The colored sections indicate the distribution into detractors, passives and promoters. The pointer indicates the NPS value.

Next, the NPS was calculated for each group by subtracting the percentage of detractors from the percentage of promoters. The group with low customer trust achieved an NPS of −68.6. The group with high customer trust achieved an NPS of 36.5, see Fig 2. The difference in NPS between the two groups is 105.1 points. The results from the Mann-Withney-U-test indicated a significant difference between the groups. The analysis of the Net Promoter Score (NPS) distributions shows significant differences between the trust groups. Subjects with low trust have a lower median NPS ($\tilde{x}$* *= 6.00) compared to subjects with high trust ($\tilde{x}$* *= 9.00). The Mann-Whitney U-test revealed a statistically highly significant difference between the groups (*U* = 55387.000, *z* = −24.674, *p* < .001). The effect size *r* = .66, which corresponds to a strong effect and indicates a substantial practical significance of the observed difference.

**Fig 3:**
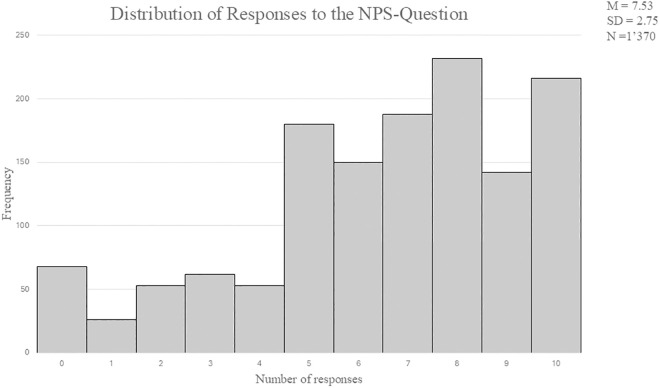
Frequency Distribution of NPS Values. The descriptive statistics for the dependent variable (NPS) indicate pronounced variation and moderate negative skewness. The mean value (*M* = 7.53) falls below the median ($\tilde{x}$ = 8.00), suggesting an asymmetric distribution. The interquartile range spans 4 points (Q1 = 6, Q3 = 10), demonstrating considerable dispersion within the central portion of the distribution.

The employment of quantile regression was undertaken to capture the heterogeneity inherent in the effects of customer trust components across varying levels of Net Promoter Score (NPS). Conventional ordinary least squares (OLS) regression procedures presuppose constant effects throughout the entire distribution, thereby potentially obscuring discernible variations in customer behavioral patterns. The residual plot analysis (S3 Fig) further indicates potential violations of the homoscedasticity assumption, as the variance of residuals appears to vary systematically across fitted values. Quantile regression facilitates the development of a more comprehensive understanding of inter-variable relationships through the examination of distinct distributional segments, rather than focusing exclusively upon central tendencies (see Fig 3. for the distribution of the NPS values), while offering greater robustness against heteroscedasticity.

The negative values for skewness (*γ₁* = –0.718) and kurtosis (*γ₂* = –0.187) further indicate a left-skewed and slightly flattened distribution (see Table 1). These characteristics provide methodological justification for employing quantile regression to model potential differences in influencing factors across various segments of the NPS distribution.

**Table 1:** Descriptive Statistics for NPS.

*N*	*M*	*SD*	Median	Q_0.25_	Q_0.75_	Min	Mas	Skewness	Kurtosis
1370	6.53	2.75	7.00	5.00	9.00	0	10	−0.72	−0.19

*Note: N = *sample size*, M = *mean*, SD = *standard deviation*, Q_0.25_ = *0.25-quantile, *Q_0.75_* = 0.75-quantile, *Min* = minimum, *Max* = maximum.

Variance Inflation Factors (VIFs) were computed for all predictor variables to evaluate potential multicollinearity concerns. All VIF values remained below the recommended threshold of 10, indicating the absence of serious multicollinearity issues within the dataset (see Table 2).

**Table 2:** Variance Inflation Factor and Tolerance Statistics.

	VIF	1/VIF
1 Ability	6.08	0.14
2 Benevolence	6.99	0.12
3 Clarity	3.06	0.19
4 Consequences	1.31	0.76
5 Continuity	5.85	0.18
6 Experience	1.37	0.16
7 Integrity	8.01	0.17
8 Joint Interests	5.15	0.73
9 Reciprocity	5.64	0.43
10 Reputation	2.34	0.33

**Note**: VIF = Variance Inflation Factor; 1/VIF = Tolerance. Tolerance values below 0.1 indicate multicollinearity.

Model fit was assessed utilizing the pseudo *R²* statistic as proposed [54]. The models demonstrated substantial explanatory power across all quantiles, with pseudo *R²* values of 0.45 (Q₀.₂₅), 0.45 (Q₀.₅₀), and 0.41 (Q₀.₇₅), indicating that the models accounted for between 41% and 45% of the variation in the conditional quantiles of the criterion variable (NPS). This suggests that the predictor variables explain a considerable proportion of variation not only at the median but also at the lower and upper extremes of the NPS distribution. Additionally, mean absolute error (MAE) ranged from 1.20 to 1.45, indicating high predictive accuracy across all models. Table 3 presents the results of the quantile regression models estimated at the 25th, 50th, and 75th percentiles of the Net Promoter Score (NPS). The analysis reveals that the effects of trust components vary across different levels of customer evaluations

**Table 3:** Quantile Regression to test the effect of customer trust on the NPS.

	*Q*_0.25_	*Q*_0.50_	*Q*_0.75_
(Intercept)	−5.92*** (−14.54)	−3.93*** (−13.95)	−1.90*** (−6.03)
Ability	0.46** (2.56)	−0.11*** (−0.90)	−0.11*** (−0.78)
Benevolence	0.00 (0.03)	0.39 (2.73)	0.24 (1.49)
Clarity	0.18 (1.31)	0.40* (3.77)	0.41 (3.43)
Consequences	−0.22** (−2.70)	−0.24*** (−4.30	−0.20** (−3.16)
Continuity	0.26 (1.50)	0.12* (1.00)	0.03*** (0.23)
Experience	0.53*** (10.54)	0.59*** (4.69)	0.46*** (3.30)
Integrity	0.21 (1.04)	0.29** (2.42)	0.44 (3.22)
Joint Interests	0.17 (1.12)	0.47*** (13.61)	0.48*** (12.33)
Reciprocity	0.49** (2.92)	0.45 (5.99)	0.38 (4.47)
Reputation	0.69*** (6.39)	0.19*** (1.99)	0.17*** (1.64)

*Note:* Results of the quantile regression model with the NPS as the dependent variable and the Customer Trust Components as independent variables. *, ** and *** denote statistical significance at the 1%, 5% and 10% levels respectively. The figures in brackets are t-values.

The components ability (*b’s: Q*_0.25 _= 0.46, *Q*_0.50 _= 0.59, *Q*_0.75 _= 0.46), experience (*b’s: Q*_0.25 _= 0.53, *Q*_0.50 _= 0.47, *Q*_0.75 _= 0.48), and reputation (*b’s: Q*_0.25 _= 0.69, *Q*_0.50 _= 0.45, *Q*_0.75 _= 0.38) demonstrate consistent positive effects across all three quartiles, indicating stable influence on NPS regardless of distributional position. Consequences (*b’s: Q*_0.25 _= −0.22, *Q*_0.50 _= −0.24, *Q*_0.75 _= −0.20) exhibit significant effects across all three quartiles, albeit negative. This suggests that increased perception of consequences for untrustworthy behavior corresponds with decreased NPS scores. Continuity (*b’s: Q*_0.50 _= 0.29, *Q*_0.75 _= 0.44) and joint interests (*b’s: Q*_0.50 _= 0.40, *Q*_0.75 _= 0.41) demonstrate positive effects at the median and upper quartile, while clarity (*b’s: Q*_0.50 _= 0.19) and integrity (*b’s: Q*_0.50 _= 0.39) exhibit significant effects only at the median. Reciprocity (*b’s: Q*_0.25 _= 0.49) achieves significance exclusively in the lower quartile, suggesting that it plays a more substantial role in preventing low customer evaluations than in enhancing high ratings. Regarding the trust component benevolence, no significant contribution to variance explanation could be detected.

## Discussion

The NPS is increasingly recognized as a paramount metric in the business sector, instrumental in directing strategies towards customer-centricity and adaptability [4]. As a critical corporate instrument, the NPS enables firms to pinpoint latent enhancement prospects [5], emphasizing the need for continual improvements [6]. A key factor influencing NPS is customer trust [5], but the exact mechanism and the hierarchy of trust components remain unclear. This study addressed this gap by examining how different trust components relate to NPS across various customer segments. From the research conducted, four major findings are revealed.

Firstly, the results demonstrate that customers with higher trust levels were more likely to be promoters, while those with lower trust were predominantly detractors. This distinction was supported by a Mann-Whitney-U-test, which revealed a statistically significant difference between the groups, confirming that higher customer trust leads to a substantially higher NPS. These findings are further substantiated by supplementary Spearman rank correlation analysis, which reveals a robust positive association between customer trust constructs and Net Promoter Score values, thereby providing convergent validity for the observed relationships. Therefore, theassumption that the NPS is driven by customer trust, can be accepted based on our findings.

Secondly, the findings from this study demonstrate substantial explanatory power of customer trust components on the NPS, with pseudo R² values of .45 (*Q*₀.₂₅), .45 (*Q*₀.₅₀), and .41 (*Q*₀.₇₅) across different quantiles of the NPS distribution. The quantile regression analysis reveals that the influence of trust components on NPS varies across different levels of customer evaluations, offering a more nuanced understanding than conventional methods. The differential significance patterns across quantiles reveal distinct trust dynamics at various NPS performance levels. At the lower quantile (*Q*₀.₂₅), only ability, consequences, experience, reciprocity and reputation demonstrate significant effects, suggesting that these components are particularly critical for preventing customer detraction and moving customers from negative evaluations. The significance of reciprocity exclusively at this lower quantile indicates that perceived fairness and mutual exchange become paramount when customer relationships are at risk of deteriorating. This is supported by empirical findings that demonstrate how reciprocal exchange processes between customers and businesses significantly enhance trust.

The study shows that direct exchange relationships – such as mutual benefits, positive reviews, and reciprocal assistance – are crucial for building trust [55], and when customers develop trust through these mutual, fair exchange processes, this trust-based reciprocity not only leads to loyalty but transforms customers into active advocates who proactively recommend the company, thereby expanding the circle of mutual trust [56]. At the median quantile (Q₀.₅₀), additional components such as clarity, continuity, integrity, and joint interests gain significance alongside the foundational elements, indicating that achieving average performance levels requires broader trust management. This suggests that achieving average levels of performance requires a more comprehensive approach to trust management – one that not only addresses functional satisfaction but also emphasizes transparency and a genuine alignment with shared values. Empirical findings indicate that trust-related factors such as perceived integrity are key drivers of the Net Promoter Score (NPS), particularly at the median level, where trust must be built across multiple dimensions of brand image [57]. Similarly, in sensitive service contexts such as health care, the willingness to recommend is strongly shaped by perceived openness, empathy, and value orientation [58]. Notably, at the upper quantile (Q₀.₇₅), clarity and integrity lose their significance, while continuity and joint interests remain influential, suggesting that the highest-performing customer relationships depend less on clarity and perceived integrity, and more on sustained engagement and shared objectives. This stands in stark contrast to previous research, which repeatedly shows that integrity and transparency are particularly important for building customer trust and brand loyalty [36]. This pattern demonstrates that achieving high NPS performance requires a comprehensive and multifaceted approach to trust building, where organizations must address a broader spectrum of trust determinants at median performance levels, while focusing on relationship continuity and mutual interests for promoter-level outcomes. The evolving significance patterns across quantiles emphasize the necessity of adaptive trust management strategies rather than uniform approaches across all customer performance segments.

The components ability, experience, and reputation demonstrate consistent positive effects across all three quantiles (25th, 50th, and 75th percentiles), indicating stable influence on NPS regardless of distributional position. Ability is established as a traditional pillar of trust, documented extensively in customer trust models as a crucial driver [17]. Empirical evidence presented by [42] attest that stellar customer experiences culminate in an amplified level of customer trust. In a correlative fashion, [59] posited a positive linkage between customer experiences and the NPS. Consequently, it is inferred that experience, when contemplated as a trust determinant, contributes positively to the NPS. In the same vein, an ascension in reputation corresponds with a noticeable positive impact on the NPS, paralleling the findings of [35]. Their research confirmed the correlation between a solid reputation and favorable NPS outcomes. Thus, it appears coherent that an enhancement in reputation would consequently precipitate an uplift in the NPS. Regarding consequences, the consistent negative effects observed across all quantiles suggest that increased perception of consequences for untrustworthy behavior paradoxically corresponds with decreased NPS scores. This counterintuitive finding warrants further investigation, as it may indicate that heightened awareness of potential negative outcomes creates anxiety that reduces customer advocacy intentions

In the research conducted by [45], it was empirically ascertained that mutual interests exert a substantially positive effect on the level of trust amongst customers. Joint Interests demonstrate positive effects at the median (*Q*₀.₅₀) and upper quartile (*Q*₀.₇₅), confirming their importance particularly for higher-performing customer relationships. Concurrently, it was noted that conflicts of interest inversely yielded a detrimental influence on the same metric of customer trust [46]. Bearing in mind the established positive correlation between customer trust and the NPS, it seems rather intuitive that shared interests would similarly have a considerable impact on enhancing the NPS.

Thirdly, the results indicate that specific trust components primarily drive the NPS. What initially may not strike as particularly intuitive is the fact that benevolence shows no significant contribution to variance explanation across any quantile, while integrity exhibits significant effects only at the median (*Q*₀.₅₀). These findings challenge conventional wisdom, as both components are typically considered fundamental to trust development. Widely recognised as pillars of trust, benevolence typically wields substantial influence upon it [17]. Similarly, integrity is deemed a ubiquitous factor of trust and generally perceived as a fundamental prerequisite for fostering customer trust [44]. The quantile regression analysis reveals that integrity’s impact is limited to the median distribution, suggesting its influence may be more conditional than previously assumed. A plausible explanation is that customers may consider benevolence and integrity as baseline expectations rather than differentiating factors. In established customer-company relationships, these components may represent necessary conditions for trust but prove insufficient for driving the recommendation behaviors captured by NPS. Customers may assume these qualities exist within positively perceived relationships, making them less predictive of advocacy intentions.

Finally, these findings have important practical implications for customer relationship management. The differential effects across quantiles suggest that organizations should adopt adaptive trust management strategies rather than uniform approaches across all customer segments. For customers at risk of becoming detractors (lower quantile), companies should focus on ability, experience, reputation, reciprocity, and carefully manage how consequences are communicated to avoid potential negative impacts. For average-performing relationships (median), a broader spectrum of trust components becomes relevant. For promoter-level relationships (upper quantile), sustained engagement through continuity and alignment of joint interests appear most critical. Given that customer trust is a key determinant of a positive NPS [13,20], understanding the specific trust components that influence NPS at different performance levels is essential for crafting targeted strategies. Surprisingly, traditional trust components such as benevolence and integrity, though frequently emphasized in trust-building efforts [60], show minimal or conditional effect on NPS. Benevolence demonstrates no significant impact across any quantile, while integrity’s influence is limited to median-performing customer relationships. In contrast, consistently performing components like ability, experience, and reputation appear to significantly contribute to a favorable NPS across all distribution segments. The negative effects of consequences across all quantiles present an intriguing finding that warrants further investigation, as it suggests that heightened awareness of potential negative outcomes may paradoxically decrease customer loyalty. This insight suggests that companies should prioritize aligning their service delivery with customer expectations, facilitating positive experiences for improved reputation, and carefully managing how consequences are communicated to avoid potential negative impacts on NPS. Additionally, companies should recognize that different trust components may be more or less effective depending on the current NPS level of their customer base, with reciprocity being particularly important for preventing low evaluations, while continuity and joint interests being more crucial for achieving high performance levels.. By focusing on these elements, businesses can enhance customer trust and, consequently, their NPS, fostering an environment of mutual benefit. These findings support the assumption that companies can increase their NPS by working on specific trust components, as evidenced by the presented results.

### Limitation and future research

This study provides valuable insights into the relationship between customer trust and NPS in the financial services sector of German-speaking Switzerland. Nevertheless, methodological and conceptual limitations emerge, offering targeted impulses for future research. The cross-sectional design captures only a snapshot in time and does not allow for conclusions regarding temporal or causal relationships. To understand the dynamics between trust and brand loyalty as reflected in NPS, longitudinal studies are needed that investigate changes across multiple waves of data collection. A methodological concern arises from the exclusive reliance on self-reported data. While NPS is internationally standardized as a self-report measure [10], the simultaneous collection of predictor and outcome variables through the same instrument increases susceptibility to common method variance. Additionally, the findings are inherently limited to participants who voluntarily engaged with the recruitment process and maintained attention throughout the survey, as the automatic filtering of incomplete responses and failed attention checks may have introduced selection effects that could influence the generalizability of results. Future studies should therefore incorporate objective or observable behavioral data – such as actual recommendations, repeat purchases, or customer retention rates – to mitigate method-related biases.

The focus on specific trust dimensions excludes other potential factors that may influence loyalty, such as emotional attachment, cultural proximity, personal value congruence, or prior brand experiences. Future research should adopt more comprehensive models that systematically integrate psychological, social, and contextual variables. The geographical restriction to German-speaking Switzerland limits the cultural generalizability of the findings. Since trust and loyalty are culturally embedded constructs [61], transferring the results to other linguistic or cultural contexts is only possible to a limited extent. Cross-cultural studies – e.g., comparing German- and French-speaking consumers or operating in international contexts – could shed light on how cultural factors shape trust and its impact on NPS. The exclusive focus on the financial sector also raises questions about the applicability of the findings to other industries [28]. Differences in trust mechanisms, regulatory intensity, and emotional customer engagement suggest that the observed relationships may be sector-specific. Future studies should therefore adopt a cross-sectoral approach and include markets such as consumer goods, tourism, or e-commerce. Finally, brand loyalty is operationalized solely through NPS as a unidimensional measure. Although this is methodologically well established, it does not fully capture the complexity of loyalty as a construct [62]. Further research should consider multidimensional loyalty concepts – e.g., using scales for affective, cognitive, and behavioral components – and relate them to differentiated trust metrics.

## Conclusions

The presented research uncovers new insights into how various trust components affect the NPS, both reinforcing and challenging existing beliefs. The implementation of quantile regression methodology has provided a more nuanced understanding of these relationships by revealing that trust components exhibit varying effects across different levels of customer evaluations. This suggests a need to reassess and potentially redirect efforts in trust-building to improve NPS. Key findings indicate that factors such as ability, experience, and reputation demonstrate consistent positive effects across all quantiles of the NPS distribution and should be central to strategy development. The strong correlation between customer trust and NPS (*r* = .789) and the substantial difference in NPS between high-trust (36.5) and low-trust (−68.6) groups demonstrate the critical importance of trust in driving customer loyalty. Focus should be on ensuring high-quality service delivery, creating seamless customer experiences, enhancing reputation, and carefully managing the communication of consequences to avoid potential negative impacts. Companies should also adopt a segmented approach, recognizing that reciprocity is particularly important for preventing low customer evaluations, while continuity and joint interests are more crucial for achieving high NPS levels. Integrating these elements can deepen customer trust and improve NPS. The finding that consequences exhibit negative effects across all quantiles presents an important area for future research and strategic consideration. Moreover, while traditional trust components like benevolence show no significant impact and integrity demonstrates only conditional effects at the median level, they may still represent basic customer expectations rather than differentiators. The quantile regression analysis has revealed that clarity’s impact is limited to median-performing relationships, suggesting that its influence may be more conditional than previously assumed.This insight highlights the need for a revised perspective on trust’s role in NPS dynamics. Overall, this study enhances the understanding of trust’s impact on NPS and suggests strategic adjustments. Future research should further explore the interplay of trust components in shaping NPS, while practitioners should use these insights to refine their strategies for NPS improvement.

## Supporting information

S1 Table Questionnaire to measure customer trust. (DOCX)

S2 Table Reliability analysis of the trust scale. (DOCX)

S3 Fig Residual Plot for Model Diagnostic. The residual plot analysis further indicates potential violations of the homoscedasticity assumption, as the variance of residuals appears to vary systematically across fitted values.
(TIF)
