# Supplementary material for: The trust–NPS correlation: The role of trust in promoting customer loyalty in Swiss financial institutions
Source: PLoS One. 2025 Nov 5;20(11):e0334423. doi: 10.1371/journal.pone.0334423 (PMC12588505; doi:10.1371/journal.pone.0334423)
Supplement: S1 Table — (DOCX) [file pone.0334423.s001.docx]

**S1 Table: Questionnaire for measuring customer trust.**

| **Key trust mechanisms and items** | **Source** |
| --- | --- |
| ***Benevolence*** | |
| <Company> cares about my well-being as a customer. | Doney & Cannon, 2026 |
| <Company> puts my needs as a customer first. | Höddinghaus, Sondern & Hertel, 2021 |
| <Company> takes care of my interests as a customer. | Mosavi & Ghaedi, 2012 |
| My interests as a customer are important to <Company>. | van Esterik-Okasneuher & can Raaij, 2017 |
| ***Integrity*** | |
| <Company> is sincere toward me as a customer. | new |
| <Company> is honest with me as a customer. | Mozafari, Weiger & Hammerschmidt, 2021 |
| What <Company> says and what <Company> does are consistent. | new |
| <Company> keeps their promises. | new |
| ***Shared Interests*** | |
| <Company> and I as a customer have the same interests. | new |
| <Company> and I as a customer are in the same boat. | new |
| <Company> can simultaneously protect my interests as a customer and their own interests. | new |
| I believe that <Company> acts in my interest as a customer. | Qui & Benbasat, 2017 |
| ***Consequences*** | |
| If <Company> behaves wrongly or unfairly, I can defend myself as a customer. | new |
| As a customer, I must accept <Company>'s behavior toward me because I have no alternative. (reverse coded) | new |
| If <Company> behaves wrongly toward me as a customer, I can fight back. | new |
| As a customer, I must accept <Company>'s behavior toward me because I can't do anything about it anyway. (reverse coded) | new |

| ***Reciprocity*** | |
| --- | --- |
| <Company> also trusts me as a customer. | new |
| <Company> and I trust each other. | new |
| As long as I as a customer give <Company> no reason to distrust me, <Company> trusts me. | new |
| The relationship between me as a customer and <Company> is characterized by mutual trust. | new |
| ***Ability*** | |
| <Company> has the necessary competencies to implement the offered products/services in high quality. | Höddinghaus, Sondern & Hertel, 2021 |
| <Company> is an absolute expert in the market. | van Esterik-Okasneuher & can Raaij, 2017 |
| <Company> is competent. | van Esterik-Okasneuher & can Raaij, 2017 |
| <Company> manages to turn their promises into actions. | new |
| ***Consistency*** | |
| I can rely on consistent performance from <Company>. | new |
| <Company> delivers the same quality every time. | new |
| <Company> offers the same high quality across all products/services. | new |
| With <Company>, I know what to expect. | new |
| ***Experience*** | |
| I know <Company> well. | new |
| I have little experience with <Company>. (reverse coded) | new |
| I have had few points of contact with <Company> so far. (reverse coded) | new |
| I have been able to gather many experiences with <Company>. | new |
| ***Reputation*** | |
| <Company> enjoys a good reputation. | Doney & Cannon, 1997 |
| <Company> makes a good impression on many customers. | Sweeney and Soutar, 2001 |
| Many people think poorly of <Company>. (reverse coded) | new |
| <Company>'s image is poor. (reverse coded) | new |

| ***Clarity*** | |
| --- | --- |
| As a customer, I have enough information to decide for or against a purchase from <Company>. | Corbitt, Thanasankit & Yi, 2003 |
| The information about <Company>'s offerings is easily understandable for me. | van Esterik-Okasneuher & can Raaij, 2017 |
| <Company> communicates clearly and distinctly. | van Esterik-Okasneuher & can Raaij, 2017 |
| The information I receive as a customer about <Company>'s products/services is unclear. (reverse coded) | van Esterik-Okasneuher & can Raaij, 2017 |

*Note*: Customer trust was measured across ten theoretically grounded trust components. The items were selected and adapted based on a comprehensive literature review of existing peer-reviewed questionnaires, with additional items developed as needed to ensure full coverage of all components. For each component, a mean score was calculated.
