# Supplementary material for: The trust–NPS correlation: The role of trust in promoting customer loyalty in Swiss financial institutions
Source: PLoS One. 2025 Nov 5;20(11):e0334423. doi: 10.1371/journal.pone.0334423 (PMC12588505; doi:10.1371/journal.pone.0334423)
Supplement: S2 Table — (DOCX) [file pone.0334423.s002.docx]

**S2 Table. Reliability analysis of the trust scale.**

| **Subscale** | **Chronbachs** $\boldsymbol{\alpha}$ |
| --- | --- |
| ability | .89 |
| benevolence | .92 |
| clarity | .76 |
| consequences | .68 |
| continuity | .89 |
| experience | .90 |
| integrity | .91 |
| joint interest | .89 |
| reciprocity | .89 |
| reputation | .82 |

*Notes:* Reliability for each customer trust subscale was assessed using Cronbach’s alpha, based on responses from *N*=1,370 end customers. Each subscale consists of four items. All Cronbach’s alpha values are acceptable (*α* > .60), indicating satisfactory internal consistency for all trust components.
