# Supplementary figures and images for: The trust–NPS correlation: The role of trust in promoting customer loyalty in Swiss financial institutions
Source: PLoS One. 2025 Nov 5;20(11):e0334423. doi: 10.1371/journal.pone.0334423 (PMC12588505; doi:10.1371/journal.pone.0334423)

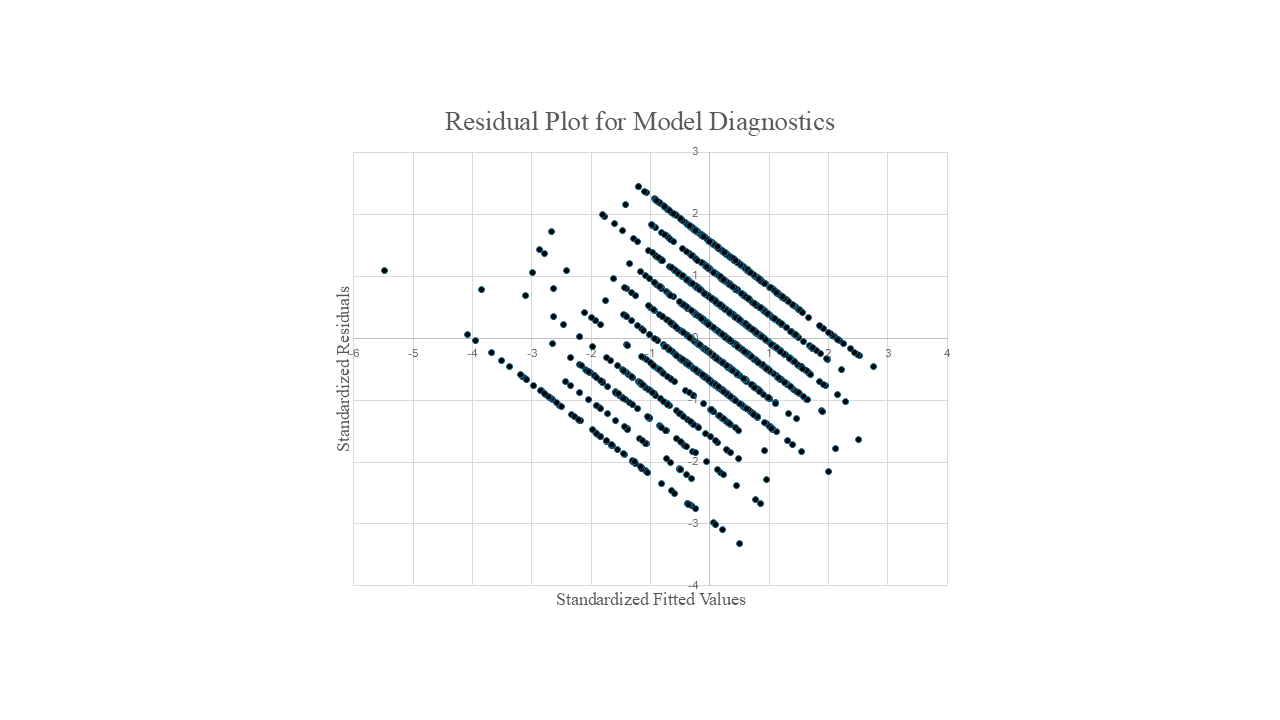

Supplement: S3 Fig — The residual plot analysis further indicates potential violations of the homoscedasticity assumption, as the variance of residuals appears to vary systematically across fitted values. (TIF) [file pone.0334423.s003.tif]
